# Acquisition and evaluation procedure to improve the accuracy of SAED

**DOI:** 10.1002/jemt.24229

**Published:** 2022-09-07

**Authors:** Zsolt Czigány, Viktória Kovács Kis

**Affiliations:** ^1^ Institute of Technical Physics and Materials Science Centre for Energy Research Budapest Hungary

**Keywords:** aberration corrected TEM, bioapatite, evaluation procedure, precision, reproducibility, SAED

## Abstract

The achievement of this work is that fine tuning of experimental and evaluation parameters can improve the absolute accuracy and reproducibility of selected area electron diffraction (SAED) to 0.1% without using internal standard. Due to the proposed procedure it was possible to reach a reproducibility better than 0.03% for camera length between sessions by careful control of specimen height and illumination conditions by monitoring lens currents. We applied a calibration specimen composed of nanocrystalline grains free of texture and providing narrow diffraction rings. Refinements of the centre of the diffraction pattern and corrections for elliptic ring distortions allowed for determining the ring diameters with an accuracy of 0.1%. We analyze the effect of different error sources and reason the achieved absolute accuracy of the measurement. Application of the proposed evaluation procedure is inevitable in case of multicomponent nanocomposites or textured materials and/or having close diffraction rings where application of automated procedures is limited. The achieved accuracy of 0.1% without internal standard is approaching that of routine laboratory XRD, and reduction of instrumental broadening due to the elaborated evaluation procedure allows for separation of close reflections, provides more reliable ring width and thus improved input parameters for further nanostructure analysis as demonstrated on dental enamel bioapatite.

## Graphical abstract

The improved reproducibility and accuracy of electron diffraction patterns without internal standard allows for reliable detection of 0.05 Å shift of peak positions and distinction and intensity analysis of 8 and 15 pixels wide diffraction rings separated by 12 pixels in case of dental enamel bioapatite.

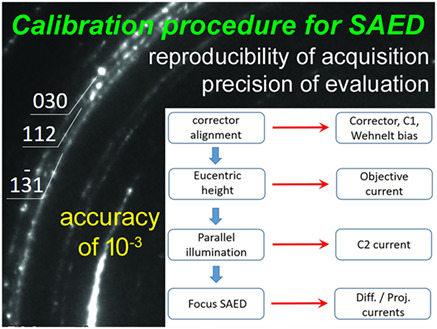

**Research highlights**

- We propose a standard acquisition procedure to improve reproducibility of SAED to 0.03%.
- We propose an evaluation procedure for multicomponent nanocomposites or textured materials where application of automated procedures is limited.
- We provide a detailed analysis of error sources and reason the absolute accuracy of 0.1% for SAED calibration.
- We demonstrate the effectivity of the procedure on bioapatite.

## 1 INTRODUCTION

Among diffraction techniques used for structure investigation (including crystal structure determination, defect structure, grain size, and texture) electron diffraction excels with its locality, however, its accuracy and reproducibility are generally considered to be lower than that of X‐ray diffraction (XRD). A conservative estimate for the accuracy and reproducibility of interplanar (d) spacing determination from electron diffraction is 1%–3% according to the famous handbook by Williams and Carter (2009). This can be improved by calibration using a sample of known lattice parameter and aiming at reproducing the acquisition conditions as pointed out in an early case study of Lodder and Berg (1974). This approach also holds the uncertainty of reproducibility between subsequent sessions (Lábár et al., 2012). The primary reason for this degree of uncertainty is that a focused diffraction pattern can be achieved by different combination of specimen height, illumination conditions and diffraction focus giving rise to different camera length (CL) values and widths of diffraction rings. Another approach for calibration can be using a substance of known lattice parameter in the analyzed specimen as an internal standard have been applied for decades but in many cases it is not practicable (Lodder & Berg, 1974). The most frequently applied internal standard is gold nanoparticles (Carvalho & Morales, 2012; Kis et al., 2017; Mugnaioli et al., 2009; Schamp & Jesser, 2005) and calibration accuracy can generally reach 0.1%. Reports about higher accuracy of 0.05% for polycrystals and 0.01% for single crystals together with a broad discussion of the subject are given by Schamp and Jesser (2005) and Carvalho and Morales (2012), respectively. Moreover, instrumental factors like astigmatism and other lens aberrations, and properties of recording medium can further deteriorate pattern quality (Carvalho & Morales, 2012; Gorelik et al., 2019; Saitoh et al., 2013).

A similar estimate like that of Williams and Carter (2009) was given for the error of camera length by Gorelik et al. (2019) in a recent study made by using an aberration corrected transmission electron microscope (TEM). They determined a systematic camera length error of 1.5%. By their consideration, this error is typical for electron diffraction and lies within the expected range. In that study they applied selected area electron diffraction (SAED) to calculate atomic pair distribution function (PDF) which can be applied for a broad variety of materials from ceramics to metallic glasses and mineralogical to organic samples. They emphasize that several interdependent parameters such as accelerating voltage, diffraction camera length, beam convergence and energy filtering will affect the SAED pattern. Furthermore, the width of diffraction rings is also influenced by the amount of astigmatism in the projector lens and the Modulation Transfer Function (MTF) of the recording medium (Gorelik et al., 2019). Since systematic study of the effect of these parameters on the data is missing, they are uncertain about the existence of TEM settings that are reproducible and suitable for calibration.

Weirich et al. (2000) faced a similar difficulty, that is, the broadening of rings due to the above mentioned parameters, which limited the chance to resolve close Bragg reflections of polycrystalline materials, like in case of tetragonal TiO_2_ anatase. Applying Rietveld analysis on SAED data, the reliability factors of their refinement were *R*_wp_ = 5.2% and *R*_B_ = 2.6%. With application of energy filtering their profile matching yielded slightly different conventional Rietveld R factors of *R*_p_ = 10%, *R*_wp_ = 10%, and *R*_exp_ = 8%, and subsequent structure refinement resulted in R factors of *R*_wp_ = 6.26% and *R*_B_ = 1.69% (Weirich et al., 2002). They predict, that improvement of acquisition of SAED in a standardized way will allow for Rietveld refinement of SAED patterns to become a routine method for quantitative analysis of polycrystalline materials which are not accessible by other methods due to their small quantity or crystallite size.

Due to the above mentioned uncertainties, many reports have been published about unreasonable accuracy that are achieved using indefinite experimental conditions. As an example for the systematic analysis of the problem, Hou and Li (2008) emphasized the importance of microscope alignment and astigmatism to minimize the effect of elliptic distortion using a non‐aberration corrected (JEOL 2010F TEM) microscope. Another systematic study of the effect of illumination parameters, specifically the current of the second condenser lens (C2) in a conventional Philips CM20 TEM was provided by Lábár et al. (2012). They found an optimum range for C2 current which allows for highest resolution of diffraction rings and were able to determine the CL with 0.3% accuracy using a nanocrystalline calibration specimen and Ditabis Imaging Plates as recording medium. Additionally, they used Process Diffraction software to compensate for elliptical distortion of the patterns.

Besides the above mentioned examples for determination of structure parameters, electron diffraction can be also used in combination with imaging techniques for nanostructure characterization. Before the period of aberration correction, electron diffractive imaging (EDI) was proposed to improve resolution in microscopy avoiding the aberrations of imaging lenses. Iterative phase retrieval procedures were proposed to reconstruct localized structures from diffraction intensity alone (Fienup, 1982; Gerchberg & Saxton, 1972; Miao et al., 1999). Some aspects of the proposed methods could be applied for early aberration corrected microscopes. Morishita et al. (2008) have developed a promising method for EDI in which wave field is reconstructed from a selected area diffraction pattern in a *Cs*‐corrected TEM for non‐localized nanostructures. Information from imaging and diffraction planes ‐ or real and reciprocal spaces—of transmission electron microscopes can be combined using iterative transformation algorithms to reconstruct the complex wave function and improve image resolution by removing residual aberrations (Zuo et al., 2011). Today, with evolution of correctors, image resolution of <1 Å can be reached routinely without using diffractive imaging.

The architecture of the aberration corrected microscopes allows for recording diffraction patterns of some nm area not only using convergent beam electron diffraction (CBED) but also with nano‐beam diffraction (NBD) (Jiang et al., 2012; Morishita et al., 2008; Uesugi, 2013; Ward et al., 2014; Wen et al., 2010; Zuo et al., 2011) enhancing the locality of the investigation. Nano‐beam electron diffraction (NBD) is a method which produces a diffraction pattern composed of spots by using a parallel illumination applied on a few nanometers wide specimen area. In aberration corrected microscopes the control of beam position is also improved (Uesugi, 2013; Yamasaki et al., 2005). Yamasaki et al. (2005) reported a beam area error of ~2 nm of a 20 nm selected area (SA) applying SAED. Uesugi (2013) achieved similar spatial resolution of strain mapping by electron diffraction using piezo specimen movement revealing ±3% strain variation. Many reports were published utilizing NBD technique to determine the structure of nm sized objects like for example crystal defects (Ward et al., 2014), quantum dots and carbon nanotubes (Zuo et al., 2011) and chiral structure of double wall carbon nanotubes (Jiang et al., 2012).

Most of the NBD studies use reflection spots belonging to the 0th order Laue zone (ZOLZ). The resulting accuracy of lattice parameter determination is approximately 0.1% for single crystal diffraction (Mahr et al., 2015; Mahr et al., 2019; Müller et al., 2012; Saitoh et al., 2013). To obtain this accuracy the correction of effects of lens imperfections is necessary. As an example Müller et al. (2012) compared 3 algorithms for elliptic distortion correction and Mahr et al. (2015, 2019) applied single disk and lattice fit techniques to determine the positions of NBD disks and applied correction for elliptical distortion using the unstrained part of the specimen as reference. They both achieved ~0.1% precision during strain mapping of semiconductor heterostructures. Saitoh et al. (2013) achieved an exceptionally low error of measurement of 0.02% for lattice parameter determination of single crystals by including high order Laue zone (HOLZ) reflection spots observed by NBD patterns. Involvement of more than 40 HOLZ reflections and correction of the distortion caused by the lens aberrations were the key factors in achieving the measurement error of 0.02%. Their method also utilizes that HOLZ line positions are sensitive to the lattice planes both parallel and perpendicular to the incident beam direction. (Analysis of ZOLZ reflections is sensitive only to the lattice planes parallel with the incident beam.) The lens‐distortion parameters were calibrated by using an NBD pattern of an MgAl_2_O_4_ spinel single crystal. The limitation of their method is that it requires strain free crystalline area. Although conventional NBD method (taking into account only ZOLZ reflections) is less accurate in lattice parameter determination, it is more suitable to investigate lattice strain in the vicinity of interfaces of hetero‐structures (where HOLZ lines cannot be clearly observed) and other nanostructures like the ones mentioned in the examples above.

Based on the examples above a significant effort was invested to exploit the advantages of aberration corrected microscopes, however, researchers do not pay sufficient attention to utilize its capabilities for powder diffraction. However, high accuracy electron powder diffraction by SAED could indeed provide average 3D structural information on nanomaterials, which is complementary to the NBD results (or HRTEM) in a similar way like powder XRD to conventional TEM but at orders of magnitude smaller scale. As acquisition of SAED patterns is fast and possible to process with existing softwares (e.g., Lábár et al., 2012, Gammer et al., 2010, Li (2007); Zou et al., 1993; see also https://www.iucr.org/resources), it is worth to invest efforts into establishing a standardized procedure for the high accuracy measurements. State‐of‐the‐art aberration corrected microscopes have an order of magnitude better acceleration voltage and lens current stability compared to conventional TEMs, which implies an order of magnitude better reproducibility and accuracy in electron diffraction. Besides the stability of acceleration voltage and lens currents, field emission gun (FEG) electron sources provide a coherent electron beam with typical energy spread of 0.7 eV which also decrease the instrumental broadening. Moreover, an additional benefit of C_S_ correction for selected area diffraction patterns is that reduced C_S_ of the objective lens helps to exclude scattered waves coming from outside of the aperture (Hirsch et al., 1977; Khouchaf et al., 2020; Lábár et al., 2012).

On the other side, aberration correction may introduce complicated higher order distortions especially at high diffraction angle where HOLZ reflections are observed, therefore, correction of distortion terms such as axial coma and 3‐fold astigmatism is required to achieve high accuracy (Saitoh et al., 2013). In case of ring diffraction patterns, which contains 3D information about the crystal lattice, it is reasonable to limit the investigation to the angular range of ZOLZ reflections and thus combined effect of the lower and higher order distortions can be parametrized as elliptical distortion of rings.

Since residual elliptical elongation of the diffraction patterns due to imperfect optics and alignments is always present, even in aberration corrected microscopes, its correction is inevitable (Gorelik et al., 2019; Mahr et al., 2019; Saitoh et al., 2013). Procedure for determination of elliptical distortion parameters was developed by Capitani et al. (2006) and similar procedures are implemented in most of diffraction processing softwares (e.g., Lábár et al., 2012; Lábár & Das, 2017; Gammer et al., 2010, Mitchell, 2008; Carvalho & Morales, 2012; Li (2007); Zou et al., 1993; see also https://www.iucr.org/resources) and also for evaluation of 4D STEM data (Savitzky et al., 2021). Note that higher order elliptical distortion may also be present and none of the above softwares treat them. Therefore, it is of fundamental importance to develop reproducible standard procedures for SAED acquisition to minimize the residual (non‐elliptical) distortions.

Moreover, phase identification of nanomaterials by electron powder diffraction is hampered not only by calibration accuracy, but also by significant instrumental contribution to line broadening, which leads to peak overlapping in case of lower symmetry materials or multicomponent samples. Full pattern fitting (Boullay et al., 2014; Song et al., 2012; Weirich et al., 2000; Weirich et al., 2002) or pair distribution function analysis (Gorelik et al., 2019) are proposed to handle this problem, however, the efficiency of these procedures (e.g., the R factor of the fitting) is limited by the accuracy of 1D line profile extracted from the ring diffraction pattern which may lead to misinterpretation of the structure. Most of the difficulties arise from the above mentioned distortions of the electron microscope lenses and from uncertainties of diffraction pattern centre determination during evaluation. Regarding the latter, several procedures exist for finding the (XY) centre coordinates of a ring diffraction pattern (Gammer et al., 2010; Lábár & Das, 2017; Mitchell, 2008; Zou et al., 1993), however, their applicability is limited by discontinuous ring/arc diffraction patterns especially where rings are located close to each other. Note that these kind of structures limit the applicability of procedures for determination of the parameters of elliptic distortion as well. The interrelation of the centre and ellipticity parameters arise further difficulties during diffraction pattern evaluation. In full profile fitting procedures the camera length, the centre and ellipticity parameters of the SAED pattern are all fitted parameters, together with structure parameters, which may involve the interdependence of calibration and structure parameters. In this paper we rather follow the concept proposed by Lábár et al. (2012) where the calibration procedure, centre refinement and correction for ellipticity are independent of the investigated structure. Moreover, we are aiming at combining it with the approach proposed by Hou and Li (2008), that is, improving the microscope alignment and providing a step‐by‐step reproducible procedure to minimize the effect of optical distortions.

In this work we propose a procedure to improve the reproducibility and accuracy of selected area electron diffraction (SAED) patterns obtained on polycrystalline materials. During acquisition of the diffraction patterns we carefully controlled the specimen height and illumination conditions by monitoring lens currents in a C_S_ corrected electron microscope. For evaluation we developed a procedure to refine the centre and elliptic distortion parameters of the SAED patterns paying special attention to patterns where discontinuous rings (texture) and overlapping rings are involved. For this purpose, we selected the most suitable calibration specimen. The precision and limitations of the method are demonstrated both on our calibration specimen and on a nanocrystalline bioapatite specimen.

## 2 EXPERIMENTAL METHODS

The electron diffraction patterns were taken in a Themis (Thermo Fisher) TEM operated at 200 kV and equipped with Cs correction in the imaging system (spatial resolution in HRTEM mode 0.8 Å). The Cs corrector consists of hexapole and quadrupole elements. The microscope is equipped with Schottky field emission gun (FEG) having an energy spread of ~0.7 eV. SAED patterns were recorded by a 4k × 4k Ceta camera using Velox software (Thermo Fisher).

Electron transparent lamellae of human dental enamel (textured nanocrystalline bioapatite) were made by a Scios2 (Thermo Fisher) focussed ion beam (FIB) system. In the final period of preparation, the energy of Ga beam was decreased to 2 keV to minimize beam damage. The crystal parameters of hexagonal structure hydroxylapatite *a* = 9.424 Å and *b* = 6.879 Å, space group *P*6_3_/*m* (ICSD26204) were used as reference.

For calibration purposes a 30 nm thick polycrystalline DC sputtered Cu film was used, which was deposited at 150°C onto a TEM grid coated with polycrystalline (2–3 layers) multilayer graphene (SPI G1000‐BA; Lot# 1190519). The Cu film deposition was performed in an ultra‐high vacuum (UHV) compatible vacuum chamber (base pressure of 6 × 10^−6^ Pa) in 0.3 Pa Ar with 3 Å/s deposition rate. The typical lateral crystallite size of the Cu film was 20–50 nm. The thin multilayer graphene film has narrow diffraction rings which were utilized for refinement of ellipticity parameters and polycrystalline Cu has non‐overlapping rings in the observed scattering angle range.

The diffraction patterns were exported in 16 bit tiff format and 1D diffraction profiles, containing intensity distribution as function of scattering angle, was obtained using Process diffraction software (Lábár et al., 2012). The software allows visual fit and refinement of centre of the pattern (X, Y), eccentricity (*ε*) and its angle (*α*) together with calibration of camera length and refinement of these parameters in case of continuous separate rings. To determine the precision of refinement steps in the evaluation procedure the peak positions were fitted using pseudo Voight function in Origin software.

## 3 RESULTS AND DISCUSSION

### 3.1 Corrector alignment

**FIGURE 1:**
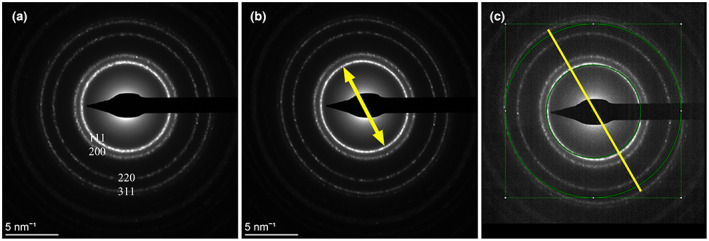
Demonstration of the effect of wrong corrector alignment on the ellipticity of diffraction rings in the SAED pattern of polycrystalline Al. (a) Circular rings can be achieved by adjustment of diffraction astigmatism, however, the central spot remains astigmatic. (b) the diffraction astigmatism was adjusted on the central spot which resulted in elliptic rings. (c) the elongation of elliptic diffraction rings (same settings as in panel b) depend on the diffraction angle: The deviation from the reference circles increase at higher diffraction angle. The yellow arrow and line indicate the direction of elliptic elongation

As a starting point of the preparation for acquiring high quality diffraction patterns in a C_S_ corrected TEM, it is important to check the basic alignment of the corrector system with respect to diffraction mode. Wrong diffraction alignment of the corrector can be recognized by having difficulties with correction of diffraction lens astigmatism and getting round diffraction rings at the same time. Moreover, diffraction angle dependent elliptic elongation of diffraction rings may be observed. Such wrong alignment is demonstrated in Figure 1. With adjustment of diffraction astigmatism circular rings can be achieved (Figure 1a), but with these settings the spot of central beam becomes astigmatic (The central spot is not shown: we used beam stop to protect the camera). In Figure 1b the diffraction astigmatism was adjusted on the central spot. In this case the rings are elliptic. In addition, the settings demonstrated in Figure 1b may give rise to diffraction angle dependent elongation of elliptic diffraction rings as can be followed by increasing deviation from the reference circles in Figure 1c at larger scattering angles. This kind of higher order distortion due to diffraction astigmatism is attributed to the quadrupole component close to the top of the corrector. Seeing such behavior, the operator is advised to contact the service specialist of the manufacturer since regular users have no access to the adjustment of the corrector element that is responsible for this kind of distortion. This basic service adjustment is important to get rid of higher order (non‐elliptical) components of optical distortions. Since the above mentioned corrector adjustment will affect other aberrations (mainly 4‐fold astigmatism (A3) and star aberration (S3)) as well, the usual image corrector alignment procedure is necessary to set all the adjustable aberration parameters below the confidence level. The latter alignment can be done by the local operator.

For polycrystalline ring diffraction patterns analyzed in this study at 200 kV it is reasonable to perform the investigation at relatively low diffraction angles to observe d spacings larger than 1 Å, which coincides with the angular range of ZOLZ reflections. After making the above alignment properly, the diffraction angle dependence of elliptic elongation in the angular range of ZOLZ reflections is negligible. For the angular range of HOLZ reflections, correction of high order distortion terms may still be necessary as advised by Saitoh et al. (2013) to achieve high accuracy.

### 3.2 Standardization of acquisition parameters

**FIGURE 2:**
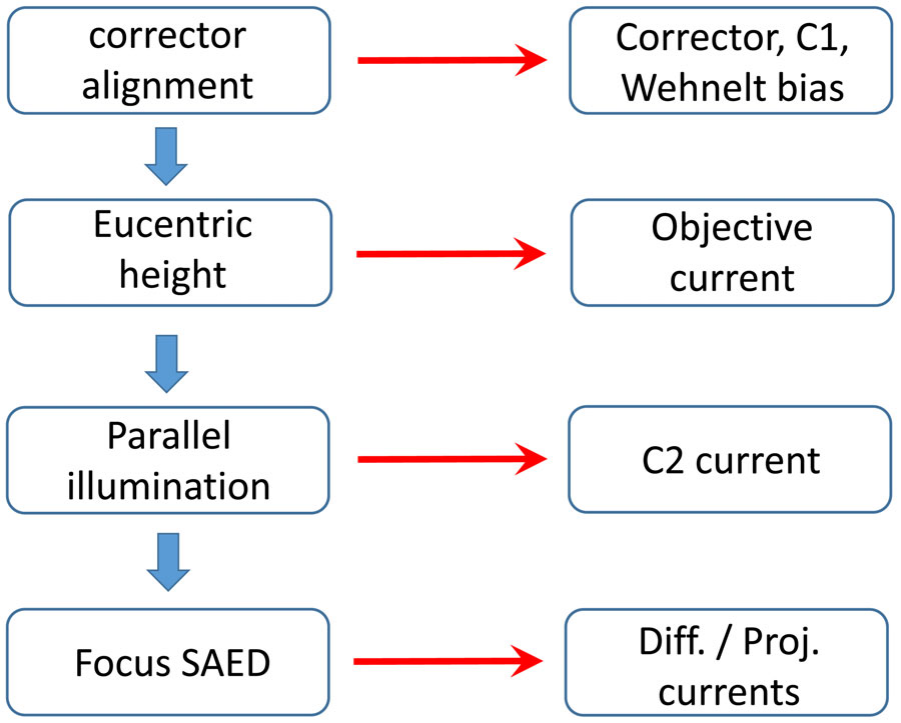
Successive steps of adjustment procedure for reproducible SAED acquisition at a given camera length. The first column contains the list of actions, the second column indicates the corresponding controlled parameters

Reproducibility of the CL of the diffraction is important both for comparison with the calibration specimen and for comparison of patterns taken with long time interval. Reproducibility can be achieved by keeping constant the illumination conditions and the specimen height ensuring the reproduction of the lens currents. The scheme of successive steps is illustrated in Figure 2.

The illumination can be standardized through the parameters of electron gun and the illumination system. The adjustable parameter of the electron gun is the bias of the Wehnelt cylinder (GunLens parameter in Themis). The spot size is related to the current of the first condenser lens. Both parameters influence the beam intensity and coherence. These parameters should be fixed and an alignment of the image corrector should be created and saved for these parameters. In our experiments we used GunLens = 3 and spot size = 6 both to ensure coherent illumination and low intensity for beam sensitive materials (e.g., bioapatite). For setting the same specimen height, the objective lens current must always be the same. In most microscopes there is a button to standardize the objective lens current (e.g., “eucentric focus” in Themis microscope) to adjust it to the eucentric specimen height. Then the specimen must be focussed mechanically by Z specimen movement and piezo Z movement if available for fine adjustment at high magnification (where lattice fringes may be observed). Later when the diffraction pattern is adjusted/focussed, further Z specimen movement must be avoided. (In some microscopes fine Z specimen movement can be assigned to focus button and attention needed to switch back its function to *focus* when the diffraction patterned is focussed.)

During investigation, the same parallel illumination should be chosen (related to the current of the second condenser (C2)). In our experiments we used 6 μm diameter of the illuminated area at 45 k magnification. With these illumination settings (i.e., C2 and GunLens = 3, spot size = 6) the SAED patterns were acquired at a dose rate of 1.53 e/Å^2^s, which is equivalent with 228 pA screen current without specimen. It is also important to use the same magnification (as was previously advised by Lábár et al. (2012)) since these parameters are interrelated (as can be followed by monitoring the lens currents). Due to the complicated interrelation of the illumination parameters, in our case no such clear tendency of the diffraction ring widths could be observed like the dependence reported in Lábár et al. (2012) for the simpler illumination system of the Philips CM20 microscope as a function of C2. In this study we used the same nominal camera length of 650 mm. Though the size of selected area (SA) aperture is not supposed to influence the CL, at the above standardized illumination conditions we used SA200 having a diameter of ~3 μm on the specimen.

As a last step before acquisition, the diffraction astigmatism and diffraction focus should be adjusted. Applying these precautions, the lens currents were the same for all experiments up to the last displayed digit indicating that the reproducibility of the experimental conditions is 1–3 × 10^−4^.

Due to possible dependence of the width of diffraction rings on the Modulation Transfer Function (MTF) of the recording medium (Carvalho & Morales, 2012; Gorelik et al., 2019; Saitoh et al., 2013) it is also advisable to acquire the SAEDs in the best available resolution (in our case 4 k × 4 k) and avoid the saturation of the camera. Of course the best if one can keep the intensity in the linear range of the sensor.

### 3.3 Evaluation of electron diffraction data

#### 3.3.1 Artifacts produced by misadjustment of centre and eccentricity

For evaluation of diffraction patterns we used Process Diffraction software (Lábár et al., 2012). Prior to calculation of 1D intensity profile from 2D ring pattern, parameters of elliptical distortion (*ε*—eccentricity and *α*—angle) should be determined using a calibration specimen and the obtained data should be transferred to the analyzed specimen. The ideal calibration specimen is polycrystalline having narrow rings, free of texture and has known structure and lattice parameter.

**FIGURE 3:**
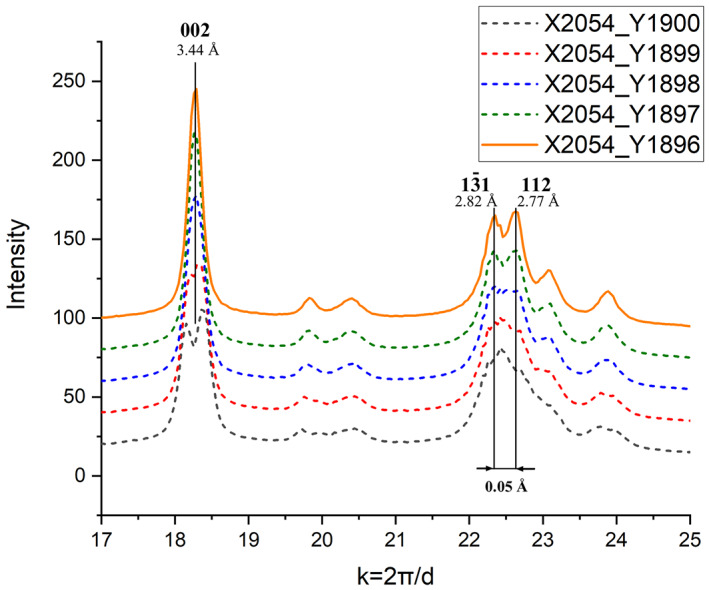
Sensitivity of intensity distribution on the misadjustment of the Centre of the diffraction pattern. When the Centre is shifted 4 pixels from the optimal value (*X* = 2054, *Y* = 1896), 002 peak splits while 1–31 and 112 peaks merge. Intensity distribution for optimal values is plotted with continuous line

The rough adjustment of the centre of the SAED pattern can be done by the visual fit of the reference ring and diffraction rings of the pattern. Wrong adjustment of the above mentioned parameters can cause split of a single diffraction ring or merge of close reflections as can be observed in the diffraction pattern of bioapatite (Figure 3), where split of 002 peak and merge of 1–31 and 112 peaks are demonstrated due to 4 pixels shift of the centre. Note that for this demonstration, the optimized ellipticity parameters are taken from the calibration specimen. Similar peak shifts and broadening were reported in Shi et al. (2013).

**FIGURE 4:**
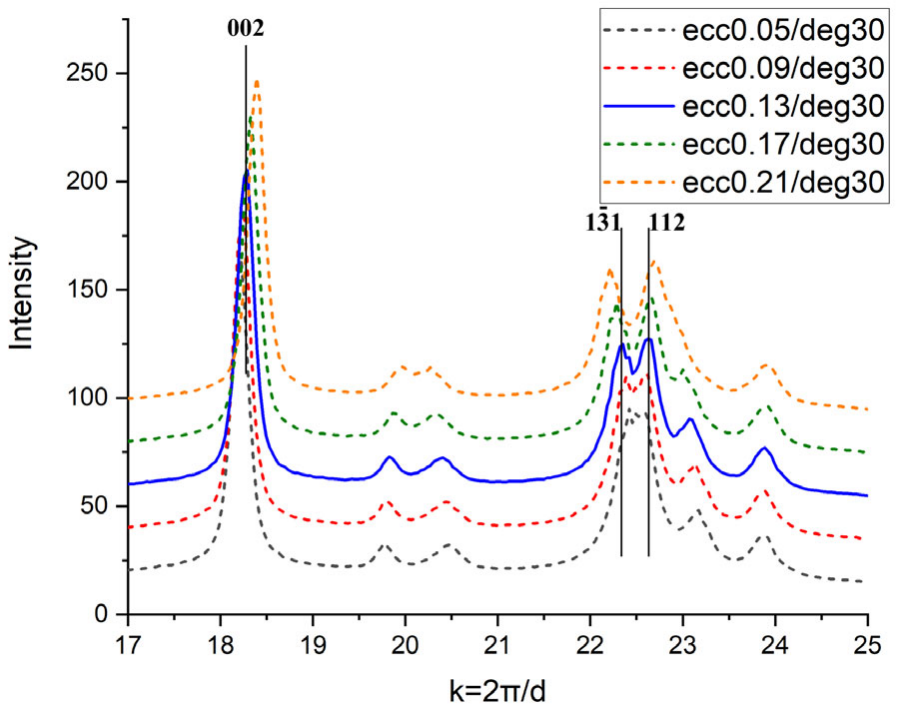
Sensitivity of intensity distribution of bioapatite on the variation of eccentricity (*ε*) compared to optimal ε value of 0.13; *α* = 30°. Peak shifts can be observed for different *ε* values compared to their nominal values. (Note that the applied ε deviations are quite large compared to the adjustment tolerance for *ε*!) Intensity distribution for optimal values is plotted with continuous line.

Furthermore, deviation from the optimum ε value slightly influence the position of Bragg peaks as shown in Figure 4 for the same peaks of bioapatite like in Figure 3. (In Figure 4, we applied the optimized center parameters for the demonstration of the effect of *ε*.) Note that the shift direction of peaks may be different for each reflection. It depends on texture, i.e. the relation of strong ring segments compared to the elongation direction of elliptical distortion. Consequently, this kind of merge and split of rings depends strongly on the grain size and texture of the actual specimen. These phenomena can be very disturbing in case of low symmetry structures like apatite or multicomponent specimens where several overlapping rings can be observed and can be misleading in case of an unknown structure. Proper determination and refinement of *X*, *Y*, *ε*, and *α* is inevitable both for accurate calibration and reliable interpretation of the diffraction pattern of the analyzed specimen. Inaccurate setting of the, *ε* and *α* parameters can be most successfully identified by visualization of the 1D intensity profile of a textureless calibration specimen which have narrow and non‐overlapping diffraction rings.

The proposed standard procedure is the following. First the calibration specimen should be evaluated by optimization of *X*, *Y*, *ε*, and *α*. The best to start the adjustment with the center since the optimum of center position is independent of the ellipticity in case of textureless calibration specimen. After optimization of *ε* and *α*, iteration of center and ellipticity parameters may follow for fine refinement. Since the structure and lattice parameter of the calibration specimen is known, the camera length can be determined after refinement of *X*, *Y*, *ε* and *α*. The calibrated CL, together with ε and α, can be transferred to the analyzed specimen.

#### 3.3.2 Adjustment of ellipticity on a calibration specimen

Inaccurate ε value can be best adjusted using a narrow peak like the 11 reflection of polycrystalline multilayer graphene by visual observation of the ring after *X*, *Y* refinement and provides an estimate for a tolerance range of ±0.01 for *ε* where the effect of further refinement is not recognizable.

The effect of *α* can be recognized in peak broadening observed by changing *α* in steps of 7.5°. The 15° deviation from the optimum value can be compensated for, but the effect of 7.5° deviation from the optimum value cannot be recognized. Due to the ideal properties of the calibration sample (continuous, separated and narrow rings) this step also can be performed using automated algorithms to obtain ε and α (see e.g., Lábár & Das, 2017). Finally, the camera length calibration can be refined using the polycrystalline Cu reflections. Typical value of CL = 736.8 ± 0.1 mm can be achieved with an accuracy/tolerance of 1.4 × 10^−4^. The refined *ε*, *α*, and CL values can be transferred to the evaluation of the analyzed specimen.

#### 3.3.3 Evaluation of the analyzed specimen

The next step after calibration is the evaluation of the analyzed specimen. The *X* and *Y* values should be refined using CL and ellipticity parameters obtained on the calibration specimen. For complex nanostructures (e.g., texture, overlapping rings etc.) *X* and *Y* can be refined by trial and error strategy. In case of bioapatite the visual observation of separation of closely spaced rings (e.g., 1–31 and 112 in Figure 3) can be utilized as a control feature during *X*, *Y* refinement (Shi et al., 2013). It is important in all steps to calculate the distribution without automatic centre refinement because the shadow of the beam stopper and possible texture of the polycrystalline sample may mislead the centre refinement algorithm. Full profile fitting can be an effective alternative of trial and error strategy in refinement of *X*, *Y* parameters (Boullay et al., 2014) even in case of textured materials.

### 3.4 Reproducibility and error of the measurement

#### 3.4.1 Reproducibility of SAED acquisition and precision of evaluation

Applying the procedure described above we were able to guarantee that we used the same lens currents and the calibrated camera length was CL = 736.7–737.0 mm which means ±0.02%–0.03% reproducibility between sessions for several months. However, the parameters of the elliptic distortion were less stable in the long run. The eccentricity of elliptical elongation changes between 0 and 0.17 and its angle variates between 0 and 30 degrees in long time range. Therefore, these parameters should always be refined for the session using a suitable calibration specimen.

Reproducibility between specimens within a session is inevitable if one wants to use an external standard for calibration. Within a session the experimental parameters are identical within the tolerance range discussed in Chapter 3.3.2 resulting a CL = 736.8 ± 0.1 mm (Err_CL_ = 1.5 × ^−4^). Typical values and tolerances for the ellipticity parameters determined on the calibration specimen are: *ε* = 0.13 ± 0.01 and *α* = 15° ± 7.5°.

To determine the precision of the evaluation and overall accuracy of lattice spacing determination on the analyzed specimen, the cumulative effect of uncertainties in centre and ellipticity refinements should be considered.

#### 3.4.2 Error of eccentricity refinement

**FIGURE 5:**
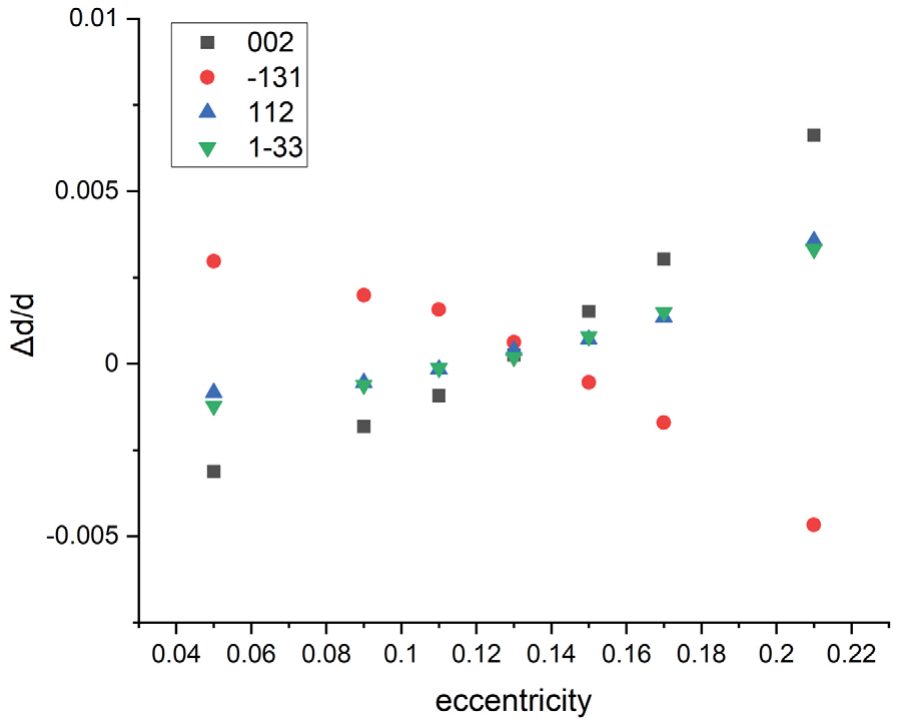
Relative difference of peak positions compared to nominal value as a function of eccentricity (*ε*) for different *hkl* peaks of bioapatite. The optimal eccentricity fit is 0.13

Figure 5 shows the value of relative peak shifts (deduced from Figure 4) for several peaks of bioapatite due to misadjustment of ε. The graph indicates small (10^−2^) relative peak shifts for large errors of *ε* (>> 0.01) and no systematic dependence on diffraction angle was observed. These data make it possible to determine the error of lattice spacing determination caused by ε misadjustment. Multiplying the highest slope of the curves (5 × 10^−2^, for 002 and 1–31 reflections) with the 0.01 tolerance of ε determination gives an error (Err_ε_) of 4 × 10^−4^, caused by uncertainty of *ε*. The observed variation caused by misadjustment of α is much smaller: Err_α_ = 1 × 10^−4^.

#### 3.4.3 Error of centre refinement

**FIGURE 6:**
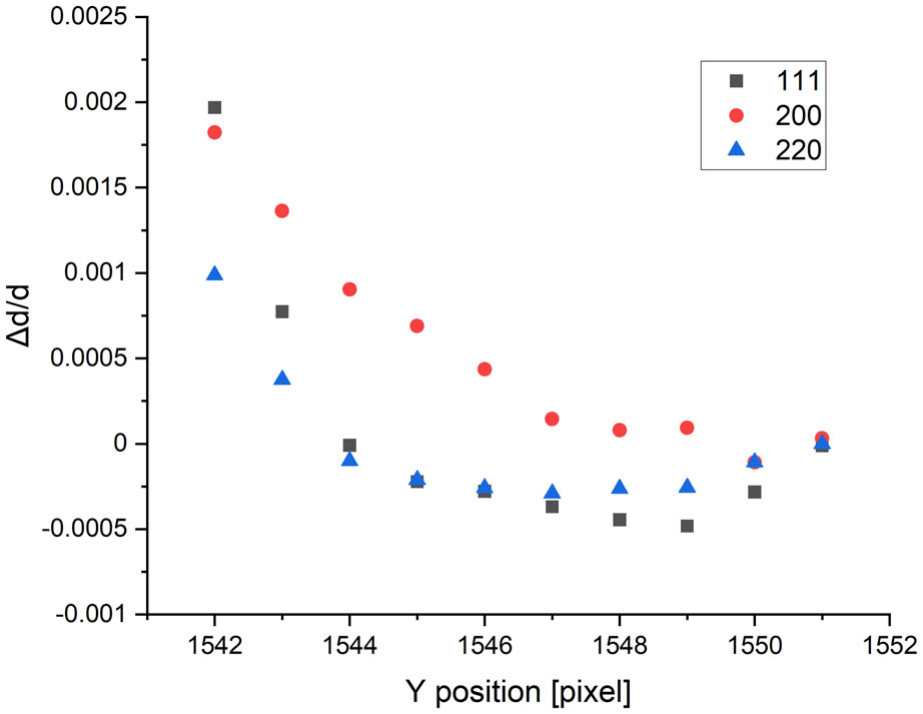
Relative difference of peak positions compared to nominal value as a function of shift of Centre in pixels for different *hkl* peaks of polycrystalline cu film

Figure 6 shows the value of relative peak shifts for several *hkl* peaks as a function of deviation from the best fit of the diffraction pattern centre (in units of pixel) for polycrystalline Cu. Assuming no texture and applying the calibrated *ε* and *α* values to compensate for elliptical distortions, the variation of one coordinate is equivalent with a replacement in any direction. The curves for different *hkl* reflections have close, but not exactly coinciding minima (where the variation due to distance from the center is minimal) which makes it possible to localize the centre within 2 pixels, which may serve as a sufficiently good starting parameter for an automatized algorithm. The scatter of the points within this range of 2 pixels is ±5 × 10^−4^, which can be considered as the error (Err_XY_) caused by uncertainty of *X* and *Y*.

The overall relative error (Err_a_) of lattice spacing determination can be calculated by

(1) $${\mathrm{Err}}_{a}^{2}={\mathrm{Err}}_{\mathrm{CL}}^{2}+{\mathrm{Err}}_{\varepsilon }^{2}+{\mathrm{Err}}_{\alpha }^{2}+{\mathrm{Err}}_{\mathrm{XY}}^{2}.$$

as a cumulative effect of the uncertainty of CL, centre and ellipticity refinements resulting an error of Err_a_ ≤7*10^−4^. The most significant contributions come from the precision of centre and eccentricity refinement.

We can conclude that the accuracy of lattice parameter measurement is better than 0.1% with the described procedure. This value was confirmed experimentally by repeated investigation of the same calibration specimen within the same session (in other words, by using the calibration specimen as analyzed specimen.)

### 3.5 Achievements—example on bioapatite

The introduction of the standard acquisition procedure for SAED resulted in ±0.02%–0.03% reproducibility of camera length between sessions. High level of reproducibility is inevitable to transfer the value measured on the calibration specimen to the analyzed specimen if calibration without internal standard is needed. Similar level (0.027%) of relative precision was claimed by Vigouroux et al. (2014) who achieved this precision by precession electron diffraction during strain mapping using 4.2 nm probe. Combined with the proposed standard calibration procedure, the absolute accuracy of that measurement could be lower than 0.06%.

The <0.1% error for lattice parameter measurement based on SAED of textured polycrystalline materials is significantly better than the generally expected values for SAED and worth of comparison with that of routine measurements on conventional laboratory XRD equipment. Previously, similar accuracy was achieved only by using internal standard. Approximately 0.1% for absolute accuracy can be routinely achieved by application of internal standard (e.g., Lábár et al., 2012; Mugnaioli et al., 2009). Similar values for measurement error were reported by processing single crystal NBD patterns as well (Mahr et al., 2015;Mahr et al., 2019), but these experiments also involve preliminary knowledge about the lattice parameters of the investigated material (= application of internal standard). Better accuracy than 0.1% with internal standard by taking into account higher order correction was reported by Schamp and Jesser (2005), Saitoh et al. (2013) and Carvalho and Morales (2012) achieving 0.05%, 0.02%, and 0.01%, respectively.

The ±0.1% error achieved by us for polycrystalline SAED on a Cs corrected TEM is 3 times better than the value given by Lábár et al. (2012) who used the same approach for analysis of polycrystalline materials elaborating a standard acquisition procedure for a non‐aberration corrected TEM. Since they also claim that 0.1% accuracy is achievable using an internal standard, the main part of their achieved absolute error is due to the transfer of CL from the calibration specimen to the investigated specimen. In other words, their accuracy mainly originate in the reproducibility of the acquisition conditions between subsequent sessions. Comparing it with our 0.03% session to session reproducibility in an aberration corrected microscope there is an order of magnitude improvement of reproducibility.

It is generally quoted that XRD and TEM are complementary techniques providing global and local information, respectively. However, in most cases the information extracted from SAED in TEM considered to be an order of magnitude less accurate. With the improvement presented here the accuracy of SAED is approaching that of routine laboratory XRD, but with 2–3 orders of magnitude better locality. Moreover, locality involves more homogenous investigated volume causing less peak broadening increasing the chance to achieve the limits of the accuracy level of the applied technique.

**FIGURE 7:**
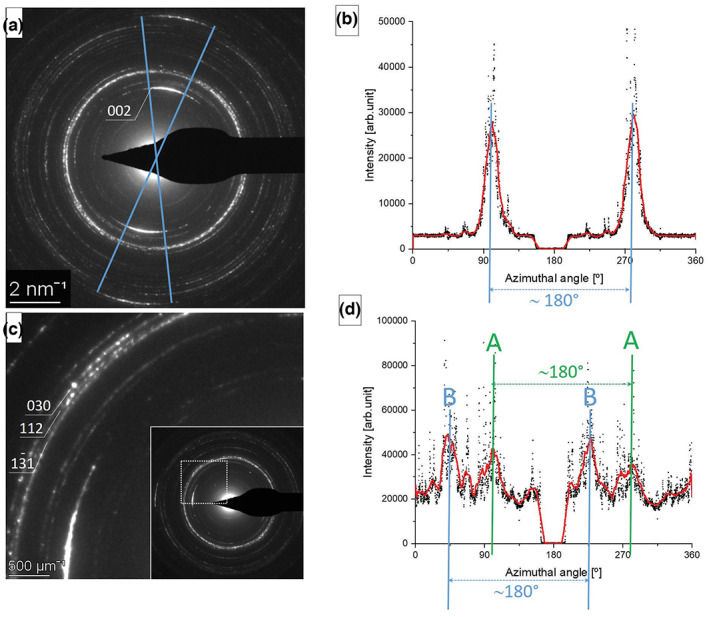
(a) A typical SAED pattern of dental enamel cross section showing strong [001] texture. (b) Azimuthally integrated intensity profile of 002 reflections in a 15 px wide ring. (c) 030 diffraction ring can be resolved (separated from 112), and (d) azimuthal intensity profile of the 030 reflections obtained after integration in an 8 px wide ring shows two sets of crystallite population A and B separated by approximately 45°

As an example, the above detailed measurement and evaluation procedure is applied on dental enamel bioapatite. Texture, its orientation and magnitude can be determined from SAED data not only for 002 (Figure 7a and b) but also for 030 diffraction ring (Figure 7c and d). The 030 ring is difficult to separate from neighboring 112 ring in routine SAED measurements (due to the small lattice spacing difference of 0.05 Å which is equivalent to 12 pixels in a 4k × 4k SAED pattern), thus adjustment of centre and elliptic distortion parameters is essential. SAED patterns of dental enamel cross section are shown of Figure 7a and c. The two SAEDs are taken from the same area, before and after rotation of the sample by 90° to avoid shadowing effect of the beam blanker. Intensity distribution along diffraction rings as function of azimuthal angle was obtained by integrating in a 15 px and 8 px narrow ring for 002 and 030 diffractions rings (12 pixels away), respectively. While Figure 7c exhibits one well defined pair of peaks separated by 180°, corresponding to [001] texture, Figure 7d shows two pairs of peaks labeled by A and B with an angular separation of approximately 45°, which indicates two sets of crystallite [030] orientation, one of them (A) corresponds to the [001] texture, the other (B) originates from another coexisting crystallite orientation population in the studied volume. These results can be discussed in comparison to synchrotron measurements (e.g., Al‐Jawad et al., 2012; Al‐Mosawi et al., 2018; Diez‐García et al., 2022), however, at higher spatial resolution and combined with high resolution imaging. Furthermore, as molecular dynamics simulations prove that variation of crystallite orientation on the sub‐micrometer scale strongly affects mechanical properties of dental enamel (Beniash et al., 2019), it is presumed that such findings will contribute to the interpretation of hardness variations measured in dental enamel cross section (Kis et al., 2021).

**FIGURE 8:**
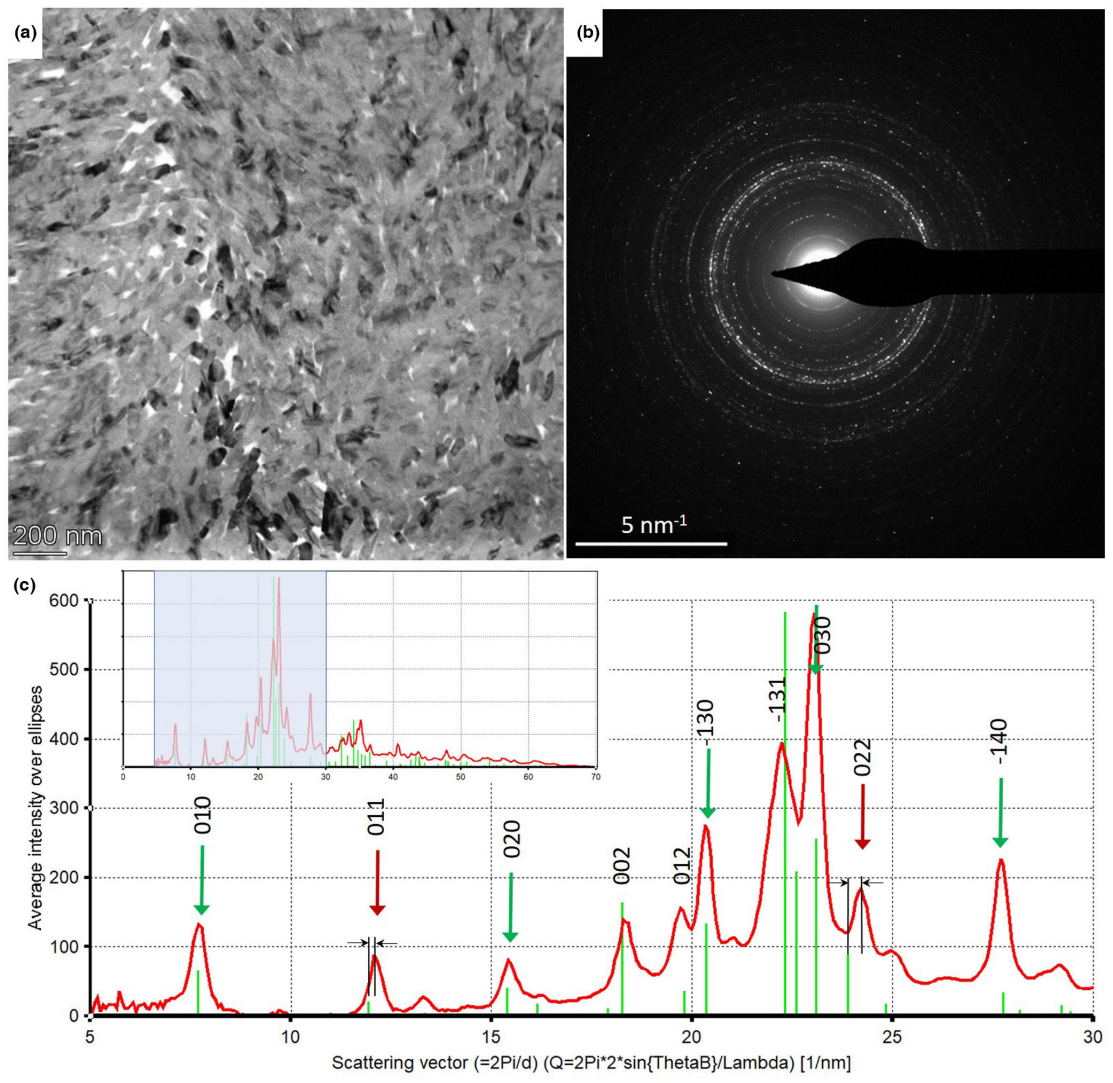
(a) TEM bright field image of dental enamel. (b) SAED pattern taken from an area of 3 μm diameter. (c) Intensity profile of SAED pattern. The main peaks are indexed according to hydroxylapatite structure (ICSD‐26204). Green arrows indicate exact coincidence of measured peaks and peaks calculated from structural data. Red arrows indicate shift of 0*kk* miller index peaks with respect to hydroxyapatite structure

As another example, a/c lattice parameter ratios for dental enamel bioapatite between 1.371 and 1.378 were obtained, which are in good agreement with X‐ray synchrotron data (Al‐Jawad et al., 2012; Raue & Klein, 2010). Additionally, in a specific area of the dental enamel cross section, systematic shift of 0*kl* reflections of ca 1.5% in terms of *d*‐value (0.05 Å) was observed (Figure 8), while the position of *hk*0 reflection coincides with literature data for inorganic hydroxyapatite (ICSD‐26204). This observation is related to the measured Mg surplus in this specific area and contributes to the interpretation of hardness properties (Kis et al., 2021). The detailed analysis of phases and interpretation of texture will be presented in a separate paper.

### 3.6 Perspectives of the presented refinements of SAED

The standardization of the acquisition parameters and careful refinement of centre and ellipticity of the SAEDs have further advantages besides the precision of calibration since the instrumental broadening of the reflections can also be reduced. This advantage extends the range of potential applications. For example, in case of single crystals, standardized acquisition and evaluation procedure improves accuracy of d values and results sharper diffraction spots. This allows for more accurate measurement of the angle of reflections in SAED patterns of single crystals in zone axes, which can be inevitable for unambiguous phase identification. (Such option of angle measurement is offered in many softwares, e.g. in Process Diffraction applied in this work). As for electron powder diffraction patterns taken from nanocrystalline materials, the proposed procedure promotes separation of close peaks which can be utilized in case of complicated (low symmetry) structures or multicomponent materials. With minimizing the instrumental contribution to peak width, more realistic estimate can be deduced for grain size by Scherrer equation. More realistic measurement of peak area can contribute to more reliable determination of phase ratio and texture.

Diffraction line profile analysis (LPA) is a popular technique to extract grain size and defect density (i.e., dislocation and twin boundary density) parameters from XRD patterns, that is, evaluated by the extended convolutional multiple whole profile (eCMWP) fitting method (Balogh et al., 2006; Ribárik et al., 2004). However, quite few attempts were made to extract so complex information from line profile analysis of electron diffraction pattern (Boullay et al., 2014; Gammer et al., 2010; Gammer et al., 2011a, 2011b). Generally, full profile fitting of SAED provided less information compared to eCMWP analysis of XRD patterns. Gammer et al. (2010), Gammer et al. (2011a, 2011b) determined grain size, Boullay et al. (2014) analyzed anisotropic crystallite sizes and shapes as well as texture using electron diffraction data. Their analysis did not extend to local strain analysis quantified in terms of defect densities like in eCMWP of XRD data. The main limiting factor is the high contribution of instrumental component to the reflections in SAED patters that can be improved further applying the presented procedure.

Quite many attempts were made to do structure refinement by Rietveld analysis of electron diffraction data (e.g., Boullay et al., 2014; Shi et al., 2013; Song et al., 2012; Weirich et al., 2000, 2002) applying a wide range of conventional (non‐aberration corrected) microscopes equipped mostly with LaB_6_ cathode and a variety of image recording media like film (Weirich et al., 2000), imaging plate (Weirich et al., 2002) and CCD (Boullay et al., 2014; Song et al., 2012). In some cases, the type and fundamental parameters of the microscope are scantly specified or completely undefined (Shi et al., 2013). In this approach the camera length, the centre and ellipticity parameters of the SAED pattern are fitted parameters together with structure parameters, which involves the mutual influence of calibration and structure parameters. These reports publish measured lattice spacings and refined parameters with unreasonable accuracy mainly in the range of 10^−4^ (Shi et al., 2013; Song et al., 2012; Weirich et al., 2000, 2002), in some cases 10^−5^ (Boullay et al., 2014). Their reliability factors can be misleading, since the high number of reported digits in the refined values may reflect only the accuracy of the mathematical fit without physical meaning. Full profile fitting algorithms allow to interpret effects of microscope alignment and evaluation procedure as structural features of the assumed phase. Precautions presented in our work to standardize the measurement procedure and improve its reproducibility utilizing the high stability of acceleration voltage and lens currents in Cs corrected microscopes and narrow energy distribution provided by FEG sources together with improvement of evaluation of SAED should be the basis to pave the way for Rietveld structure refinement and diffraction line profile analysis as well.

## 4 CONCLUSIONS

By careful control of specimen height and illumination conditions it was possible to reach a session to session reproducibility of 0.03% for camera length in SAED measurements. The key factor in achieving this reproducibility is the reproduction of the lens currents, which was achieved in an indirect way by controlling parameters like specimen height, Wehnelt bias, spot size and illumination angle and taking care of the general alignment of the objective corrector. The parameters to compensate for the elliptic distortion of lenses can also be determined by using a suitable calibration sample. An ideal calibration specimen is composed of nanocrystalline grains of a known phase without texture and produce narrow diffraction rings. Using such calibration specimen, the quality of the parameters can be judged by visual observation of the intensity distribution and subsequent refinement of the parameters by trial and error strategy. Although the SAED pattern is affected by several interrelated parameters, refinements of the centre of the diffraction pattern and corrections for lens distortions (ellipticity of rings) allowed for determining the ring diameters with a relative error of less than 0.1%. The achieved accuracy of 0.1% for lattice spacing measurement without internal standard is compatible with that of routine laboratory XRD, however, with orders of magnitude better locality. Decrease of instrumental contribution also allows for separation of close reflections, provides narrower ring width allowing for more reliable grain size determination (Scherrer) and determination of phase ratio and texture. Moreover, such improvement in acquisition and evaluation of SAED may pave the way for electron diffraction based Rietveld structure refinement and diffraction line profile analysis as well. The advantages of the described procedure are most beneficial and inevitable for low symmetry structures, textured and multicomponent materials when the diffraction pattern has many Bragg reflections with non‐uniform intensity and overlapping rings.

## AUTHOR CONTRIBUTIONS

**Zsolt Czigány:** Conceptualization; data curation; formal analysis; investigation; methodology; visualization; writing – original draft. **Viktória Kovács Kis:** Conceptualization; data curation; formal analysis; funding acquisition; investigation; methodology; project administration; visualization; writing – review and editing.

## CONFLICT OF INTEREST

The authors declare no potential conflict of interest. The authors alone are responsible for the content and writing of the paper.

## Data Availability

The data that support the findings of this study are available from the corresponding author upon reasonable request.
